# Clinical heterogeneity of Kabuki syndrome in a cohort of Italian patients and review of the literature

**DOI:** 10.1007/s00431-021-04108-w

**Published:** 2021-07-07

**Authors:** Francesca Di Candia, Paolo Fontana, Pamela Paglia, Mariateresa Falco, Carmen Rosano, Carmelo Piscopo, Gerarda Cappuccio, Maria Anna Siano, Daniele De Brasi, Claudia Mandato, Ilaria De Maggio, Gabriella Maria Squeo, Matteo Della Monica, Gioacchino Scarano, Fortunato Lonardo, Pietro Strisciuglio, Giuseppe Merla, Daniela Melis

**Affiliations:** 1 Pediatric Unit, Translational Medicine Department, Federico II University Hospital, Naples, Italy; 2 Medical Genetics Unit, San Pio Hospital, Benevento, Italy; 3 Pediatric Unit, Department of Medicine, Surgery and Dentistry “Scuola Medica Salernitana”, (Salerno), Baronissi, Italy; 4 Pediatric Unit, San Giovanni di Dio e Ruggi d’Aragona University Hospital, Via San Leonardo, 1 – 84131 Salerno, Italy; 5 Medical Genetics Unit, Cardarelli Hospital, Napoli, Italy; 6 Department of Pediatrics, AORN Santobono-Pausilipon, Napoli, Italy; 7 Division of Medical Genetics, IRCCS Casa Sollievo della Sofferenza, San Giovanni Rotondo, Foggia, Italy

**Keywords:** Kabuki syndrome, Autoimmunity, Brain anomalies, Neurological features

## Abstract

Kabuki syndrome (KS) is a well-recognized disorder characterized by postnatal growth deficiency, dysmorphic facial features, skeletal anomalies, and intellectual disability. The syndrome is caused by KMT2D gene mutations or less frequently KDM6A gene mutations or deletions. We report a systematic evaluation of KS patients from Campania region of Italy; data were also compared with literature ones. We collected data of 15 subjects (8 males and 7 females with age range 10–26 years; mean age 16.9 years) with confirmed diagnosis of KS, representing the entire cohort of patients from Campania Region. Each patient performed biochemical testing and instrumental investigation. Neuro-intellectual development, cranio-facial dysmorphisms, and multisystem involvement data were collected retrospectively. For each category, type of defects and frequency of the anomalies were analyzed. Our observation shows that KS patients from Campania region have some particular and previously underscored, neurological and immunological findings. We found high prevalence of EEG’s abnormalities (43%) and MRI brain abnormalities (60%). Microcephaly resulted more common in our series (33%), if compared with major cohorts described in literature. Biochemical features of immunodeficiency and autoimmune diseases including thyroid autoimmunity, polyserositis, and vitiligo were observed with high prevalence (54.5%). Low immunoglobulins levels were a frequent finding. Lymphocyte class investigation showed significantly reduced CD8 levels in one patient.

*Conclusions*: These data confirm great heterogeneity of clinical manifestations in KS and suggest to introduce further clinical diagnostic criteria in order to perform a correct and precocious diagnosis.

**Table:** 

**What is Known** • *Kabuki syndrome is characterized by growth deficiency, dysmorphic facial features, skeletal anomalies, and intellectual disability* • *Immune dysfunction is a common finding but autoimmune diseases are rarely seen* • *Neurological features are common*	
**What is New** • *Some particular facial features could help gestalt diagnosis (hypertelorism, broad nasal bridge, micrognathia, tooth agenesis, cutaneous haemangiomas and strabismus)* • *Higher prevalence of autoimmune disorders than previously reported* • *Particular neurological features are present in this cohort (EEG and MRI brain abnormalities)*

**Supplementary Information:**

The online version contains supplementary material available at 10.1007/s00431-021-04108-w.

## Introduction

Kabuki syndrome (KS, OMIM # 147920 and 300867) was firstly described by Niikawa and Kuroki [1–3] and, over the years, has become a well-recognized multiple congenital anomaly/intellectual disability (ID) disorder.

The incidence of KS is about 1/32,000 of live births [4]. The lysine (K)-specific methyltransferase 2 family (KMT2 A-E), originally named the myeloid/lymphoid or mixed-lineage leukemia (MLL1-5) proteins, regulates the expression of genes involved in embryogenesis and development. *KMT2D* (12q13.12, also known as *MLL2*, OMIM *602113) was the first gene associated with KS [5–9], and most KS patients bear *KMT2D* gene mutations. Additionally, a minority of patients have mutations or deletions of *KDM6A* (Xp11.3, OMIM *300128), which takes part of the same transcription complex as *KMT2D* [10–16]. Potential genetic defects remain unknown in about 30% of patients clinically diagnosed with KS [17].

KS is included in the chromatinopathies, a group of hereditary disorders caused by abnormalities of chromatin regulation, determined by variants in the various genes encoding for the components of the epigenetic machinery. Neurological impairments or ID are common features, though these conditions are characterized by clinical heterogeneity [18]. The widespread of next-generation sequencing methods improved diagnosis and expanded knowledge about these disorders [19].

Niikawa et al. [1, 3] initially defined five cardinal features of KS, consisting of postnatal growth deficiency, dysmorphic facial features, skeletal anomalies, persistent fingertip pads, and ID (typically in the mild to moderate range) [20, 21].

The consensus diagnostic criteria for KS were created by an international group of experts in 2018 [22–28].

Here, we perform a systematic evaluation of a cohort of patients representing the entire medical record of patients from Campania region and compared reported data with the ones reported in the literature [24–32].

## Subjects and methods

### Subjects

Data of 15 subjects with KS, representing the entire cohort of patients from Campania region of Italy were collected. All the patients were followed up in Medical Genetics Units. The study was approved by the Medical Ethics Committee of “Federico II” University of Naples.

In this retrospective study cranio-facial dysmorphisms, neuro-intellectual development, and multisystem involvement data were collected. For each category, the type of defects and the frequency of the single anomalies were analyzed.

Auxological, neurologic, ophthalmologic, ear-nose-throat (ENT), and rheumatologic evaluations were performed.

Laboratory investigation for baseline thyroid profile, autoantibodies for autoimmune thyroiditis, screening for celiac disease and serum immunoglobulins were also recorded.

Lymphocyte class investigation was performed in 5 patients.

Auditory brainstem response (ABR), electroencephalogram (EEG), magnetic resonance imaging (MRI) of brain and cervical spine, echocardiocolor-Doppler, and abdominal ultrasound were also performed.

### Molecular analyses

Clinical diagnosis was confirmed in all patients performing molecular studies on DNA extracted from peripheral blood lymphocytes. Genomic DNA was extracted from fresh and/or frozen peripheral blood leukocytes of patients and their available family members using an automated DNA extractor and commercial DNA extraction Kits (Qiagen, Germany). Mutation screening of all 54 coding exons of the *KMT2D* (MIM #602113, NM_003482.3) gene and 29 coding exons of the KDM6A (MIM #300128, NM_021140.3) gene was performed by PCR amplification and direct sequencing as reported [33].

## Results

In this study, 15 patients, 8 males and 7 females with age range 10–26 years (average 16.9 years), have been included; 13 patients present heterozygous mutations in *KMT2D* (86.7%); and 2 patients present heterozygous mutations in *KDM6A* (13.3%). Almost all, except one, reported patients had de novo variants. One patient inherited a *KMT2D* variant from the affected mother, who presents a mild phenotype characterized by typical facial features (long palpebral fissures, lower palpebral eversion, epicanthus) and joint pain, without involvement of other systems.

**Fig. 1:**
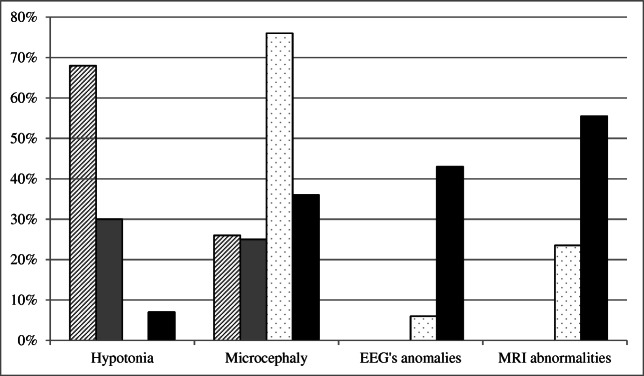
Prevalence of nervous system abnormalities in patients of present cohort and in the patients reported in the literature from Matsumoto et al. [21] (white bars with diagonal lines pattern), Wessels et al. [23] (gray bars), Banka et al. [24] (white bars with dots), this paper (black bars). EEG electroencephalography, MRI magnetic resonance imaging

**Fig. 2:**
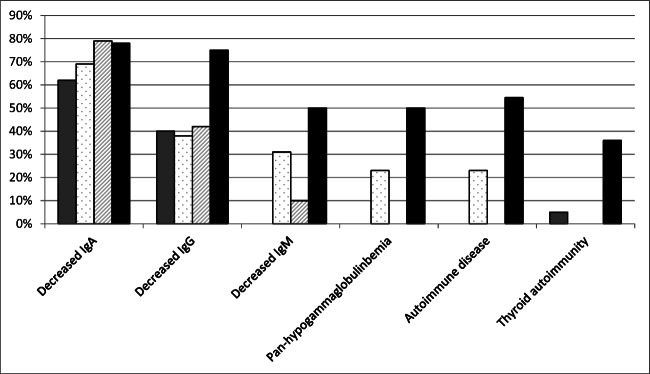
Prevalence of immune system abnormalities in patients of this cohort and in patients reported in the literature from Stagi et al. [30] (gray bars), Lindsey et al. [26] (white bars with dots), Hoffman et al. [32] (white bars with diagonal line pattern), this paper (black bars)

**Fig. 3:**
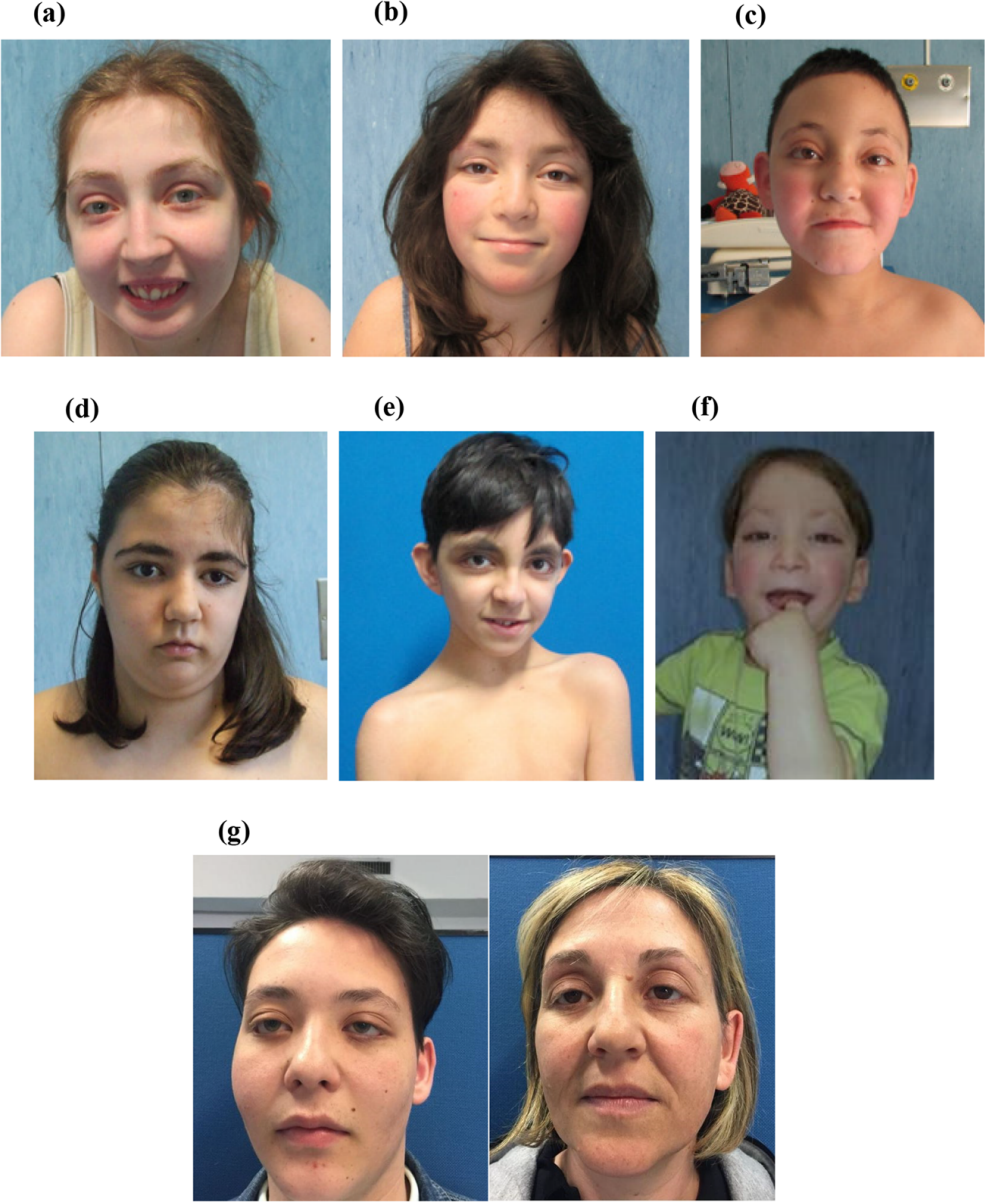
Facial fetaures in some of KS patients reported in this paper; the code is the same reported in Tables S1–S2. **a** P5. Long palpebral fissure, palpebral eversion, hypertelorism, arched eyebrow with thinning of lateral third, malformed and prominent ear, broad nasal root, short columella, thin lips, microretrognathia. **b** P11. Long palpebral fissure, palpebral eversion, epicanthus, thinning of lateral third of eyebrow, malformed and prominent ear, broad nasal root, thin lips, micrognathia. **c** P9. Long palpebral fissure, palpebral eversion, strabismus, ptosis, exophtalmos, epicanthus, arched eyebrow with thinning of lateral third, malformed and prominent ear, short columella, thin lips, high forehead. **d** P3. Long palpebral fissure, palpebral eversion, ptosis, strabismus (surgical correction), malformed ear, broad nasal root, anteverse nostrils, thin upper lip, low neck implant. **e** P15. Long palpebral fissure, eversion of third lateral, arched and thick eyebrow with thinning of lateral third, malformed and prominent ear, broad nasal root, short columella, depressed nasal tip, thin upper lip, down lip corners, micrognathia, high forehead. **f** P14. Long palpebral fissure, palpebral eversion, ptosis, arched eyebrow with thinning of lateral third, malformed and prominent ear, broad nasal root, short columella, thin lips with lip pit, micrognathia, high forehead. **g** P7 and his mother. Long palpebral fissure, lower palpebral eversion, epicanthus and prominent ear in a patient of our cohort (on the left) and his affected mother (on the right)

Main clinical features of patients, compared to literature records, are summarized in Tables 1, 2, 3, 4, 5, and 6 and Figs. 1, 2, and 3. Data in tables are separately shown for children (0–16 years, *n* = 8) and adults (> 16 years, *n* = 7). Detailed informations for each patient are available in Tables S1–S2 (see Supplement). Only significant results are reported in the text.

**Table 1:** Craniofacial features of patients of this paper compared with those reported in literature (Matsumoto et al. [21], Wessels et al. [23], Banka et al. [24], Cheon et al. [25], Lindsley et al. [26], White et al. [27], Schrander-Stumpel et al. [29])

	Wessels et al. [23]	Matsumoto et al. [21]	White et al. [27]	Schrander-Stumpel [29]	Banka et al. [24]	Cheon et al. [25]	Lindsley et al. [26]	Present study		
								Adults, *N* 7	Children, *N* 8	All, *N* 15
Characteristic face		115/115 (100%)		20/20 (100%)		12/12 (100%)	13/13 (100%)	7/7	8/8	15/15 (100%)
Long palpebral fissure	286/300 (95%)	135/136 (99%)	25/27 (93%)			12/12 (100%)		7/7	8/8	15/15 (100%)
Lower palpebral eversion	269/300 (90%)	132/143 (92%)	18/27 (67%)			12/12 (100%)		6/7	7/8	13/15 (87%)
Epicanthus		63/138 (46%)						3/6	2/7	5/13 (38%)
Ptosis	27/240 (11%)	26/52 (50%)	15/27 (56%)					2/6	4/7	6/13 (46%)
Hypertelorism								3/6	1/7	4/13 (31%)
Arched eyebrows	237/300 (79%)	165/193 (85%)	10/27 (37%)			7/12 (58%)		4/5	6/8	10/13 (77%)
Thinning of lateral third								5/7	7/8	12/15 (80%)
Malformed ear		87/100 (87%)	18/27 (67%)			11/12 (92%)		6/7	8/8	14/15 (93%)
Prominent ear	237/300 (79%)	145/172 (84%)	17/27 (63%)			11/12 92%		4/7	8/8	12/15 (80%)
Preauricular dimple/fistula		40/180 (22%)		4/20 (20%)						
Short columella		72/78 (92%)						2/6	6/8	8/14 (57%)
Broad nasal root	22/240 (9%)					12/12 (100%)		6/6	6/8	12/14 (86%)
Depressed/bulbous nasal tip	170/240 (71%)	106/128 (83%)	19/27 (70%)			8/12 (66%)		6/6	3/8	9/14 (64%)
Anteverse nostrils								2/6	2/8	4/14 (29%)
Abnormal dentition	145/300 (48%)	116/171 (68%)	12/27 (44%)		11/17 (65%)			6/6	3/3	9/9 (100%)
Oligodontia				16/16 (100%)				0/6	1/3	1/9 (11%)
Dental agenesis								5/6	0/3	5/9 (55.5%)
Dysodontiasis								1/6	1/3	2/9 (22%)
Odontoma								1/6	0/3	1/9 (11%)
Diastema								1/6	0/3	1/9 (11%)
Malocclusion								1/6	0/3	1/9 (11%)
High-arched palate	132/300 (44%)	64/89 (72%)			7/20 (35%)			4/4	5/7	9/11 (82%)
Cleft palate/lip and palate/lip	132/300 (44%)	68/196 (35%)	6/27 (22%)	9/20 (45%)	3/20 (15%)	7/12 (58%)	6/13 (46%)	0/4	3/7	3/11 (27%)
Lower lip pit		4/15 (27%)	5/27 (19%)					0/4	1/8	1/12 (8%)
Micrognathia	38/240 (16%)	37/93 (40%)		19/20 (95%)				3/4	5/7	8/11 (73%)
Low posterior air line		38/67 (57%)						1/3	0/4	1/7 (14%)
High forehead								0/3	5/5	5/8 (62.5%)
Cutaneus hemangioma								2/6	2/5	4/11 (36%)

**Table 2:** Neurological features of patients of this paper compared with those reported in literature (Matsumoto et al. [21], Wessels et al. [23], Banka et al. [24], Cheon et al. [25], Lindsley et al. [26], White et al. [27], Schrander-Stumpel et al. [29])

	Wessels et al. [23]	Matsumoto et al. [21]	White et al. [27]	Schrander-Stumpel [29]	Banka et al. [24]	Cheon et al. [25]	Lindsley et al. [26]	Present study		
								Adults, *N* 7	Children, *N* 8	All, *N* 15
Intellectual disability (IQ < 70)	262/300 (87%)	157/188 (84%)	27/27 (100%)	20/20 (100%)	20/20 (100%)	11/12 (92%)	9/9 (100%)	6/7	7/8	13/15 (87%)
Hypotonia	72/240 (30%)	32/47 (68%)	19/27 (70%)			9/12 (75%)		1/7	0/8	1/15 (7%)
Neonatal hypotonia		23/81 (28%)		18/20 (90%)	12/16 (75%)			0/7	0/7	0/14
Microcephaly	75/300 (25%)	47/179 (26%)	4/27 (15%)	8/20 (40%)	13/17 (76%)			1/7	4/8	5/15 (33%)
Neonatal microcephaly				5/20 (25%)	0/8			0/2	1/1	1/3 (33%)
Seizure	24/300 (8%)	33/194 (17%)	5/27 (19%)	4/20 (20%)	4/17 (23.5%)	2/12 (17%)		2/5	0/2	2/7 (29%)
EEG’s anomalies					1/17 (6%)			1/5	2/2	3/7 (43%)
Early infancy feeding difficulties			16/27 (59%)	18/20 (90%)	11/16 (69%)					
Dysarthria			5/27 (19%)							
Delayed myelination					1/17 (6%)					
MRI abnormalities					4/17 (23.5%)			2/5	4/5	6/10 (60%)
Enlarged ventricles					2/17 (12%)			1/5	1/5	2/10 (20%)
Corpus callosum anomaly								0/5	1/5	1/10 (10%)
Brain atrophy/White matter hypoplasia/cerebellar vermis hypoplasia		2/51 (4%)	1/27 (4%)		3/17 (18%)			1/5	0/5	1/10 (10%)
Ischemia outcomes								0/5	1/5	1/10 (10%)
Empty sella								1/5	0/5	1/10 (10%)
Pituitary microadenoma								0/5	1/5	1/10 (10%)

**Table 3:** Skeletal features of patients of this paper compared with those reported in literature (Matsumoto et al. [21], Wessels et al. [23], Banka et al. [24], Cheon et al. [25], Lindsley et al. [26], White et al. [27], Schrander-Stumpel et al. [29])

	Wessels et al. [23]	Matsumoto et al. [21]	White et al. [27]	Schrander-Stumpel [29]	Banka et al. [24]	Cheon et al. [25]	Lindsley et al. [26]	Present study		
								Adults, *N* 7	Children, *N* 8	All, *N* 15
Joint laxity	124/240 (52%)	58/78 (74%)	16/27 (59%)	18/20 (90%)	13/17 (76%)	2/12 (17%)		4/4	3/4	7/8 (87.5%)
Dermatoglyphic abnormalities		76/79 (96%)								
Presence of fingertip pad	245/300 (82%)	170/190 (89%)		20/20 (100%)	11/14 (79%)	10/12 (83%)		6/6	8/8	14/14 (100%)
Skeletal abnormality		142/162 (88%)	17/27 (63%)				10/13 (77%)	6/6	8/8	14/14 (100%)
Brachydactyly (V)	186/300 (62%)	135/170 (79%)	13/27 (50%)	29/20 (100%)	7/16 (44%)	12/12 (100%)		3/6	4/7	7/13 (54%)
Clinodactyly (V)		56/112 (50%)						2/6	5/7	7/13 (54%)
Short middle phalanx (V)		60/76 (80%)						0/6	1/7	1/13 (8%)
Short metacarpus		18/51 (35%)						1/6	0/7	1/13 (8%)
Short metatarsus								1/6	0/7	1/13 (8%)
Cone-shaped epiphysis		6/47 (13%)						0/6	0/7	0/13
Coarse carpal bone		8/48 (17%)						0/6	0/7	0/13
Scoliosis		58/168 (35%)	3/27 (11%)					4/4	3/6	7/10 (70%)
Vertebral anomalies (sagittal cleft or vertebral body or other)		20/55 (36%)	1/27 (4%)					1/4	3/6	4/10 (40%)
Rib anomaly		10/55 (18%)								
Spina bifida occulta		11/59 (19%)								
Pilonidalsinus/sacral dimpling		5/6 (83%)				5/12 (42%)				
Hip dislocation/dysplasia	34/300 (11%)	32/178 (18%)	5/27 (19%)	7/20 (35%)		3/12 (25%)		1/4	1/4	2/8 (25%)
Valgus knee								2/4	1/4	3/8 (37.5%)
Foot deformity (flat foot)		13/55 (24%)	1/27 (4%)					2/4	2/4	4/8 (50%)
Craniosynostosis			1/27 (4%)

**Table 4:** Growth and endocrine features of patients of this paper compared with those reported in literature (Matsumoto et al. [21], Wessels et al. [23], Banka et al. [24], Cheon et al. [25], Lindsley et al. [26], White et al. [27], Schott et al. [28], Schrander-Stumpel et al. [29]). IUGR intrauterine growth retardation, SGA small for gestational age

	Wessels et al. [23]	Matsumoto et al. [21]	White et al. [27]	Schrander-Stumpel [29]	Banka et al. [24]	Cheon et al. [25]	Lindsley et al. [26]	Schott et al. [28]	Present study		
									Adults, *N* 7	Children, *N* 8	All, *N* 15
Short stature (-2.0 SD)	201/300 (64%)	75/136 (55%)		14/20 (70%)		7/12 (58%)		8/18 (44%)	2/7	2/8	4/15 (27%)
Polyhydramnios									1/7	4/7	5/14 (36%)
IUGR/SGA									2/7	1/7	3/14 (21%)
Postnatal growth deficiency							10/13 (77%)				
Hypothyroidism				1/20 (5%)					1/3	1/5	2/8 (25%)
Hyper-TSH									1/3	1/5	2/8 (25%)
Neonatal hypoglycemia									1/7	0/7	1/14 (7%)
Cronic hypoglycemia			2/22 (9%)		6/16 (37.5%)				0/5	2/5	2/10 (20%)
Hyperinsulinism			1/27 (4%)						0/5	1/5	1/10 (10%)
Obesity/ Overweight		11/58 (19%)							4/7	0/7	4/14 (29%)
Premature thelarche		13/46 (28%)	3/27 (11%)	7/13 (54%)					0/5	2/5	2/10 (20%)
Hypogonadism									1/5	0/5	1/10 (10%)
Hypogenitialism									1/5	0/5	1/10 (10%)
GH deficiency		1/58 (2%)		2/20 (10%)				5/18 (27.8%)	2/5	1/5	3/10 (30%)
Delayed puberty											
Cryptorchidism		18/75 (24%)		2/7 (29%)					2/5	3/5	5/10 (50%)
Diabetes mellitus			1/27 (4%)								
Generalized hirutism		7/61 (11%)	2/22 (9%)		5/15 (33%)

**Table 5:** Immunological abnormalities of patients of this paper compared with those reported in literature (Matsumoto et al. [21], Wessels et al. [23], Stagi et al. [30], Banka et al. [24], Cheon et al. [25], Lindsley et al. [26], Lin et al. [31], Hoffmann et al. [32], White et al. [27], Schrander-Stumpel et al. [29])

	Wessels et al. [23]	Matsumoto et al. [21]	White et al. [27]	Hoffman et al. [32]	Schrander-Stumpel [29]	Banka et al. [24]	Lin et al. [31]	Stagi et al. [30]	Cheon et al. [25]	Lindsley et al. [26]	Present study		
											Adults, *N* 7	Children, *N* 8	All, *N* 15
Infection in regions including the middle ear and upper airway tract	114/240 (48%)	73/116 (63%)	14/27 (52%)		20/20 (100%)			51/59 (86%)	8/12 (66%)	9/13 (69%)	0/6	2/5	2/11 (18%)
Pneumonia								15/59 (25%)					
Decreased IgA				15/19 (79%)				39/63 (62%)		9/13 (69%)	3/4	4/5	7/9 (78%)
Decreased IgG				8/19 (42%)				23/58 (40%)		5/13 (38%)	3/3	3/5	6/8 (75%)
Decreased IgM				2/19 (10%)						4/13 (31%)	2/3	2/5	4/8 (50%)
Features of Immunodeficiency (pan-hypogammaglobulinemia)						5/15 (33%)				3/13 (23%)	2/3	2/5	4/8 (50%)
< CD8 lymphocytes							0/13				1/2	0/3	1/5 (20%)
< CD4 lymphocytes							1/13 (8%)				0/2	0/3	0/5
Autoimmune diseases										3/13 (23%)	4/6	2/5	6/11 (54.5%)
Thyroid autoimmunity								2/36 (5%)			2/6	2/5	4/11 (36%)
Arthritis								1/36 (3%)			0/6	0/5	0/11
Vitiligo								8/36 (22%)			1/6	0/5	1/11 (9%)
Polyserositis											1/6	0/5	1/11 (9%)
Celiac disease								1/36 (3%)					
Crohn’s disease								1/36 (3%)					
Sclerosing cholangitis								1/36 (3%)					
Autoimmune hemolytic anemia								7/36 (19%)					
Idiopatic thrombocytopenia								20/36 (55.5%)					
Leukopenia								2/36 (5%)					
Neutropenia								3/36 (8%)

**Table 6:** Multisystem involvement of patients of this paper compared with those reported in literature (Matsumoto et al. [21], Wessels et al. [23], Stagi et al. [30], Banka et al. [24], Cheon et al. [25], Lindsley et al. [26], White et al. [27], Schrander-Stumpel et al. [29]). AoCa aortic coarctation, ASD atrial septal defect, PDA persistent ductus arteriosus, PFO patent foramen ovale, VSD ventricular septal defect

	Wessels et al. [23]	Matsumoto et al. [21]	White et al. [27]	Schrander-Stumpel [29]	Banka et al. [24]	Stagi et al. [30]	Cheon et al. [25]	Lindsley et al. [26]	Present study		
									Adults, *N* 7	Children, *N* 8	All, *N* 15
Strabismus	65/300 (22%)	54/152 (36%)	3/25 (12%)		6/16 (37.5%)		5/12 (42%)		2/6	2/7	4/13 (31%)
Exophtalmos									0/6	2/7	2/13 (15%)
Myopia									0/6	1/7	1/13 (8%)
Corneal leukoma									1/6	0/7	1/13 (8%)
Fundus oculi anomalies (atrophy)									1/6	1/7	2/13 (15%)
Retinal pigmentation		1/29 (3%)									
Blue sclerae	50/240 (21%)	38/124 (31%)		14/20 (70%)	1/16 (6%)						
Hearing loss		48/180 (27%)	8/27 (30%)	9/20 (45%)	2/16 (12.5%)		3/12 (25%)		2/3	2/3	4/6 (67%)
Chronic otitis									1/3	1/3	2/6 (33%)
Congenital heart defects	112/300 (37%)	103/247 (42%)	16/27 (59%)	5/20 (25%)	9/15 (60%)		9/12 (75%)	9/13 (69%)	2/5	8/8	10/13 (77%)
PFO									0/5	2/8	2/13 (15%)
ASD			2/27 (7%)		4/15 (27%)				0/5	2/8	2/13 (15%)
VSD			5/27 (18.5%)	3/20 (15%)	1/15 (7%)				1/5	5/8	6/13 (46%)
Bicuspid aortic valve			3/27 (11%)	2/20 (10%) *coA/bic*	1/15 (7%)				1/5	0/8	1/13 (8%)
Aortic coarctation			4/27 (15%)	2/20 (10%) *coA/bic*	1/15 (7%)				0/5	3/8	3/13 (23%)
Aortic dilatation/dysplasia				1/20 (5%)					1/5	1/8	2/13 (15%)
PDA			3/27 (11%)						0/5	2/8	2/13 (15%)
Pulmonary stenosis					2/15						
Bronchial anomalies									0/6	1/5	1/11 (9%)
Kidney/urinary tract malformation	70/300 (23%)	41/145 (28%)	10/27 (37%)	5/20 (25%)	2/12 (17%)		4/12 (33%)	9/13 (69%)	3/5	3/5	6/10 (60%)
Recurrent urinary tract infections						12/59 (20%)					
Pyelectasis			1/27 (4%)						2/5	0/5	2/10 (20%)
Renal cysts			1/27 (4%)						1/5	1/5	2/10 (20%)
Renal hypoplasia or dysplasia			2/27 (7%)						0/5	1/5	1/10 (10%)
Ectopic kidney			2/27 (7%)						1/5	1/5	2/10 (20%)
Fused kidney					1/12				0/5	1/5	1/10 (10%)
Double kidney district									1/5	1/5	2/10 (20%)
Vescicoureteral reflux			4/27 (15%)		1/12				1/5	0/5	1/10 (10%)
Hypospadias			1/27 (4%)								
Inguinal hernia									0/6	1/5	1/11 (9%)
Gastrointestinal malformation		2/33 (6%) 4/74 (5%)	5/23 (22%)					2/13 (15%)	0/1	0/1	0/2
Diaphragmatic eventration-hernia			3/27 (11%)	3/20 (15%)							
Ano-vestibular fistula			1/27 (4%)								
Anterior anus			3/27 (11%)								
Gastro-esophageal reflux			10/27 (37%)								
Hepatic abnormality (neonatal hepatitis)			1/22 (4.5%)

### Characteristic facial features

Most of the typical facial features of KS, such as long palpebral fissures 15/15 (100%), lower palpebral eversion 13/15 (87%), and arched eyebrows 10/13 (77%) with thinning of the lateral third 12/15 (80%), were present in almost all patients. Ear anomalies, micrognathia, and broad nasal root were very common. Cleft lip or palate was reported in 3/11 (27%) whereas high-arched palate was frequent 9/11 (82%). None of the patients showed full lower lips, nodules, or pits, although this is a frequent feature in KS patients. Abnormal dentition was recorded in 6/6 adults and 3/3 children, specifically dental agenesis 5/9 (55.5%); 3 children presented, respectively, eruptive cyst, dysodontiasis, and oligodontia; an adult patient presented dysodontiasis and maxillary odontoma. Finally, 4/11 (36%) patients showed cutaneous haemangiomas: two patients in frontonasal region and two patients in sacral region (Table 1).

### Neurological abnormalities

*Intellectual disability* (intellectual quotient, IQ < 70) was found in 87% of cases.

Mild (IQ 50–69) and moderate (IQ 35–49) ID was observed in 6/15 (40%) and in 3/15 (20%) patients, respectively. In 4/15 (27%), an unspecified degree was recorded. None had severe disability (IQ < 34) and 2 had normal intellective function.

Intellectual function does not change over time. Nevertheless, a patient showed an IQ improvement (from 43, with WPPSI scale at the age of 6 years, to 61, with WISC-III scale at the age of 7 years); this difference could be explained by the diffident behavior of the patient that probably influenced the first evaluation. No sufficient data were available for the evaluation of a progression of behavioral problems. Rare findings, present in three different patients, include speech delay (1/15), aggressive behavior (1/15), and severe posttraumatic stress disorder with psychotic episodes and visual hallucinations (1/15).

Hypotonia was reported in one adult patient; neonatal hypotonia has not been reported. Microcephaly was present in 5/15 (33%), with neonatal onset only in one patient.

Two patients presented seizures, one pharmacologically treated; three patients showed EEG anomalies without clinical manifestations.

Brain MRI showed anomalies in 6/10 patients: modest increase of CNS liquoral spaces (2/10), upper parietal gyrus reduction, corpus callosum abnormalities, pituitary microadenoma, partial empty sella, and consequences of CNS ischemia in one patient, respectively (Table 2 and Fig. 1).

### Skeletal abnormalities

Skeletal abnormalities were reported in 14/14 patients (100%): brachydactyly 7/13 (54%), fifth finger clinodactyly 7/13 (54%), scoliosis 7/10 (70 %), vertebral abnormalities 4/10 (40%) (2 cases of L3 vertebral cleft, one case of butterfly vertebra and one case of C7 apophysis malformation), fingertip pads 14/14 (100%). Skeletal alterations also interested hip, knee and feet (flat foot, hip dislocation, and valgus knee) (Table 3).

### Growth and endocrine system involvement

#### Growth

Harmonic short stature (height less than − 2.0 SD) was present in 27% (4/15); in three patients, height corresponded to − 2.0 SD. Mean height in the 7 adult patients (5 female and 2 male) was 155.3 cm, in particular 153 cm (− 1.62 SD) in females and 161.1 cm (− 2.32 SD) in males. Short stature was present in both adult male patients; one of them was diagnosed for growth hormone (GH) deficiency but parents refused therapy; the other one showed a familiar short stature. Growth hormone deficiency was investigated, and GH deficit was detected in 3/10 patients. Parents of one patient refused GH therapy; his height was 157 cm at the age of 22 years (− 2.94 SD). Two of them, one male and one female, underwent recombinant growth hormone therapy: one had a good response and reached a normal stature, while the other still shows short stature. GH deficiency was diagnosed in the female patient when she was 6 years old (height 105.5 cm, − 2.02 SD). The patient underwent GH treatment from the age of 6 to the age of 15.75 years (height was 152 cm, − 1.6 SD). GH deficiency in the male patient was diagnosed when he was 13 years old (height 135.6 cm, SD − 2.79). GH therapy is still ongoing, and his height at the age of 15.67 years was 149.5 cm (− 2.9 SD). No adverse effect was reported for both patients. Intrauterine growth restriction was reported in two cases and polyhydramnios in five pregnancies. One patient was born small for gestational age and two late-preterm.

#### Endocrine system

The study of thyroid function showed high TSH levels in 2/8 and autoimmunity in 4/11, but only two of these presented hypothyroidism. Recurrent hypoglycaemia was reported in two patients, just in one case in neonatal period. Hyperinsulinism was present in one child, no patients showed diabetes mellitus. Obesity or overweight were reported in 4/14 patients (29%), all adults. Cryptorchidism was present in five patients, and one of them underwent orchidopexy at age 11 years. Hypogonadism and hypogenitalism are rarely reported (Table 4).

### Immune system involvement

Autoimmune markers and/or diseases were detected in 6/11 cases: thyroid autoantibodies in 4 patients, vitiligo in one patient, and periodic fever and polyserositis in another patient.

Some patients with KS (2/11) also underwent recurrent ear and respiratory infections.

Low immunoglobulin levels were a frequent finding: decreased IgA were observed in 7/9; decreased IgG in 6/8; decreased IgM in 4/8; severe immunodeficiency with pan-hypogammaglobulinemia in 4/8 patients.

Lymphocyte class investigation was normal with the exception of one patient showing significantly reduced expression of CD8^+^ T cells (6% vs normal range 19–29%, average 23%), according with the trend of lymphocyte reduction described in the literature (Table 5).

### Multisystem involvement

Ophthalmologic examination showed strabismus in four patients, exophthalmos in two, myopia in one, corneal leukoma in one, and *fundus oculi* abnormalities in two (optical disc atrophy and bulging in the retinal nasal area).

Hearing loss appears to be common (4/6): three patients showed conductive hearing impairment, and the other one mixed hearing loss. Chronic otitis was reported in 2/6 patients.

Congenital heart disease (CHD) was reported in 10/13 patients. The most common were septal defects: ventricular defects 6/13; atrial defects 2/13; patent foramen ovale 2/13. We also reported three children with aortic coarctation, two with persistent arterial duct, one with bicuspid aortic valve, one with aortic valve dysplasia and one with aortic dilatation.

Urogenital abnormalities were present in 60% of KS patients: pyelectasis (2/10), renal cysts (2/10), double kidney district (2/10), abnormal kidney position (2/10), renal hypoplasia or dysplasia (1/10), fused kidney (1/10), and vesicoureteral reflux (1/10) were observed (Table 6).

## Discussion

Kabuki syndrome is a well-recognized multiple congenital anomaly/ID disorder, mainly characterized by dysmorphic facial features, dermatoglyphic abnormalities, postnatal growth deficiency, and ID; congenital malformations can also be present [20, 21].

Our observation shows that KS patients from Campania region of Italy have some peculiarity.

We detected high prevalence of specific facial features, such as micrognathia, hypertelorism, broad nasal bridge, tooth agenesis, cutaneous haemangiomas, and strabismus.

Tooth abnormalities were present in all patients of our cohort, in particular tooth agenesis, abnormal tooth shape, and size (pitted incisors and truncated tooth roots). *KMT2D* and *KDM6A* are expressed in the dental epithelium of human tooth germs, thus confirming their roles in tooth development [34–36].

Cutaneous hemangiomas in our cohort are present in 4/15 patients (26 %), while prevalence in general population is reported between 4.5 and 9.9% [37, 38]. Association between KS and cutaneous haemangiomas has never been reported in literature, whereas in our cohort, it is well represented.

Minor variants such as brachidactyly, clinodactyly, and joint laxity, included among diagnostic criteria, are actually quite nonspecific. On the other hand, persistent foetal fingertip (100% of present cases) is a very peculiar feature, even if not pathognomonic, since other syndromes share this feature (Pitt–Hopkins syndrome, FG Opitz–Kaveggia, 2q37 microdeletion, and fetal alcohol syndrome ) [39–42].

The most frequent ophthalmologic anomaly reported in literature is strabismus (36%) [20–27, 29], comparable to our cohort (31%). We also recorded a considerable presence of *fundus oculi* abnormalities (15%), outlining the importance of ophthalmological examination.

Middle ear infections occur in approximately 70% of patients and can lead to conductive hearing loss and speech delay [34]. Hearing impairment (mostly conductive) appears to be common in our cases (67%). Delayed speech was also reported in one patient with conductive hearing loss.

Urogenital abnormalities are reported in 30–40% of KS patients [24] and include hydronephrosis, abnormal kidney position, renal hypoplasia or dysplasia, and fusion defects [16]. These anomalies showed a high frequency (60%) in our patients.

Rare findings reported in two pediatric patients in this paper are as follows: bronchial isomerism and bronchiectasis in one; left pulmonary artery hypoplasia and thymic ectopia in another.

Scoliosis could also strengthen the diagnostic suspicion, in particular if associated with a vertebral malformation [43].

Several neurological involvements are reported in KS patients, including hypotonia, seizures, behavioral problems, and intellectual disability [44–47].

Several studies on epilepsy in KS reported that patients were likely to present with focal seizures and focal EEG abnormalities, generally with favorable outcome. High prevalence of epilepsy in KS patients without brain abnormalities was previously reported [44]. In agreement with the literature, in our cohort, 29% of patients presented seizures. A relevant data was the high prevalence of EEG anomalies, namely pointed waves, reported in 43% of cases and rarely described in literature. Interestingly, both patients (2/2) with *KDM6A* mutations showed sporadic pointed waves and epilepsy with paroxysm.

Most KS patients have normal CNS imaging, even if brain atrophy and organic structural lesions have been reported [45, 46]. On the other hand, MRI abnormalities were described with high frequency in our cohort (60%), in particular slight increase of CNS liquoral spaces (20%). Microcephaly resulted more common in our series, if compared with literature [21–27, 29].

Neurodevelopmental and behavioral problems have been extensively reported in KS [47]. Previous reports indicated a wide range of IQ, with specific deficits in motor abilities, in linguistic domains, in phonological and oromotor functions; behavioral skills seem to be fairly preserved. In the present cohort, ID (IQ < 70) was one of the primary characteristics of KS, in mild to moderate range, and behavior problems were reported in two patients.

Congenital heart disease is described in literature in 50–75% of the patients [48, 49]. Next-generation sequencing in fetuses with CHD showed pathogenic variants in *MYH6* and *KMT2D* [48]. In the present report, CHD were described in all children and in 40% of adults.

Patients with KS are also vulnerable to infections, including those affecting middle ear and upper airway tract. Recurrent infections, mostly affecting the upper respiratory tract, were recorded in 18% of our patients. Immune impairment is a common finding in KS, since correct histone-3 methylation patterns are essential to achieve modifications in Ig genes required for B cell development and function [50–53]. Approximately half of the patients present with common variable immune deficiency (CVID)-like characteristics. Concerning to healthy individuals, the numbers of memory B cells are reduced, with difficulty to generate or maintain specific antibody responses and long-term immunological memory [31, 32, 54, 55].

Lower serum IgG, IgA, or/and IgM levels have been scored in 10–79% of KS patients [55, 56]. In the current cohort, we observed hypogammaglobulinemia in several patients and reduced CD8 levels in one patient.

Autoimmune diseases are rarely seen in patients with KS [57, 58] but were documented in 54.5% of our patients.

Mutations in *KMT2D* gene are highly recurrent and occur early during tumorigenesis in diffuse large B cell lymphoma and follicular lymphoma. These findings suggest that, in KS, loss of *KMT2D* function could lead to impairment of cell maturation [50]. A significant reduction in memory B and T cells has been documented in an entire cohort of 12 KS patients with *KMT2D* heterozygous variants [31, 32, 54]. Furthermore, a reduced generation of memory cells can be based on the lack of a delayed hypersensitivity response (including purified protein derivative PPD and candidin), as documented in a Brazilian cohort of KS patients [54]. These data can help us to explain the variable occurrence (interindividual and temporal) of dysgammaglobulinemia that can increase the risk of infections or autoimmune diseases. So, the study of lymphocytes can be considered a useful tool to identify asymptomatic subjects who can develop autoimmune disorders.

Regarding endocrinological disorders, postnatal GH deficiency is reported in KS patients, while rare findings include hypothyroidism, diabetes insipidus, primary ovary dysfunction, abnormal pituitary findings on magnetic resonance images, hyperinsulinism, and hypoglycemia [59–65]. In our cases, short stature (height < − 2SD) is reported in 4 patients and 3 patients showed height corresponding to − 2SD; GH deficiency was diagnosed in 3/10 patients. It is noteworthy that short stature was present in both adult male patients, one with GH deficit and the other with familial short stature. Considering that the prevalence of GH deficiency is only 1% in the general population and 2% in one of the major reviews, it seems very high in our series. GH replacement therapy has many beneficial effects on KS children, including a significant improvement in joint hypermobility, suggesting a direct effect of GH on connective tissue [60, 61].

High levels of TSH, with thyroid hormone deficiency were recorded in 2 patients. Similar data are described in a single case report [66].

In our series, 20% of the patients showed premature thelarche; rare findings included hypermenorrhea, micropolycystic ovary, ginecomastia, hypogonadism, and hypogenitalism. High prevalence of cryptorchidism has been recorded in our cases when compared to literature.

More than 50% of KS patients reported in the literature are overweight or obese, during childhood or adolescence [16, 20–23]. In our series, overweight or obesity was present only in adult patients and three adolescents, instead, presented generalized poor growth.

Patients with KS can present with hypoglycemia, which can be transient or persistent [63–65]. One of our patients presented with neonatal hypoglycemia; chronic hypoglycemia was detected in 20% of the patients and one of them had hyperinsulinemia. KS patients with *KDM6A* variants may be at higher risk for neonatal hyperinsulinemic hypoglycemia than those with *KMT2D* variants [63, 64]. As hyperinsulinemic hypoglycemia is one of the most common causes of persistent hypoglycemia in KS patients, a high degree of suspicion is needed for early diagnosis and appropriate management.

Regarding genotype-phenotype correlation, it has been reported that patients with *KMT2D* variants show a significantly higher frequency of short stature, typical facial features, persistent fetal finger pads, renal abnormalities, and feeding problems compared with patients with *KDM6A* variants [7, 10, 16, 67–70]; conversely, *KDM6A* variants are associated with variable phenotypes, ranging from typical KS to milder clinical presentations [11–15]. Patients with *KDM6A* variants seem to have hypotonia and feeding difficulties during infancy, poor postnatal growth, and short stature.

Developmental delay and learning disabilities are generally moderate to severe in boys and mild to moderate in girls with *KDM6A* mutations [15], as expected for X-linked disorders, but it has recently been described a female patient with *KDM6A* variant showing typical facial features, severe ID, short stature, CHD, recurrent infection, and Chiari malformation [13].

In our cohort we found that patients with *KDM6A* variants showed a more severe clinical presentation. We observed two patients carrying *KDM6A* de novo variants. The first one, a 26-year-old woman, showed short stature, tipical dysmorphic features, ogival palate, maxillary odontoma and dysodontiasis, moderate ID, EEG and MRI anomalies, polycystic ovary disorder with hypermenorrea, autoimmune hypothyroidism, mild dilatation of the renal pelvis, and scoliosis with C7 apophysis malformation. The second patient was a 21-year-old woman, showing peculiar features: long palpebral fissures with eversion of the lateral third of the lower eyelids, sparseness of eyebrows’ lateral sides, ptosis of the left eye, strabismus, hypodontia, fetal pads, malformed ears, micrognathia, brachidactyly, mild ID, behavior disorder, seizures, and anterior pituitary hypoplasia. In both cases, there was no CHD. Thus, our experience suggests that *KDM6A* phenotype has moderate-severe manifestations of disease that persist even in adulthood. In particular, we underline the predominantly neurological phenotype of *KDM6A* mutations, in which epilepsy, seizures, or EEG anomalies seem much more frequent.

## Conclusion

In conclusion, we confirm that KS is characterized by a great heterogeneity of clinical manifestations and suggest to take into consideration further clinical diagnostic criteria as an aid to perform a correct and more precocious diagnosis. Some dysmorphic features very common in our series, such as hypertelorism, broad nasal bridge, micrognathia, tooth agenesis, cutaneous haemangiomas, and strabismus, could be added to the signs allowing a gestalt diagnosis.

We also outline the multisystem involvement of KS and the need of a multi disciplinary team involved in the follow-up program, in order to allow a precocious diagnosis and treatment:the team should include neurologist, endocrinologist, ophthalmologist, ENT specialist, orthopedic, immuno-rheumatologist, cardiologist, dentist, nephrologist, gastro-hepatologist, and surgeon. Indeed, disease-specific treatment is probably on the way.

## Supplementary information

ESM 1 Table S1-2. Individual data of KS patients reported in this paper (AGA, antigliadin antibodies; anti-tTG, transglutaminase antibodies; AoCa, aortic coarctation; ASD, atrial septal defect; IQ, intelligence quotient; IUGR intrauterine growth retardation; OFC, occipitofrontal circumference; PDA, persistent ductus arteriosus; PFO, patent foramen ovale; SD, standard deviation; VSD, ventricular septal defect; VUR, vesicoureteric reflux) (DOCX 37 kb)

## Abbreviations

CHD: Congenital heart disease
CNS: Central nervous system
EEG: Electroencephalogram
ID: Intellectual disability
IQ: Intellectual quotient
KS: Kabuki syndrome
MRI: Magnetic resonance imaging
