# Supplementary material for: Clinical heterogeneity of Kabuki syndrome in a cohort of Italian patients and review of the literature
Source: Eur J Pediatr. 2021 Jul 7;181(1):171–87. doi: 10.1007/s00431-021-04108-w (PMC8760211; doi:10.1007/s00431-021-04108-w)
Supplement: Supplementary file 1 — Table S1-2. Individual data of KS patients reported in this paper (AGA, antigliadin antibodies; anti-tTG, transglutaminase antibodies; AoCa, aortic coarctation; ASD, atrial septal defect; IQ, intelligence quotient; IUGR intrauterine growth retardation; OFC, occipitofrontal circumference; PDA, persistent ductus arteriosus; PFO, patent foramen ovale; SD, standard deviation; VSD, ventricular septal defect; VUR, vesicoureteric reflux) (DOCX 37 kb) [file 431_2021_4108_MOESM1_ESM.docx]

**Table S1 Individual data of presently reported patients with Kabuki syndrome**

| **Patient** | **Sex** | **Age** | **Gene** | **Type of mutation** | **Inheritance** | **Exon** | **Variant** | **AA change** | **Palpebral/ ocular** | **Eyebrow** | **Ear** | **Nasal** | **Dentition** | **Palate** | **Lip** | **Micrognathia** | **Hair/ forehead** | **Hands/ feet** |
| --- | --- | --- | --- | --- | --- | --- | --- | --- | --- | --- | --- | --- | --- | --- | --- | --- | --- | --- |
| P1 | F | 26 | *KDM6A* | Missense | NA | 17 | c.2326G>T | p.Asp776Tyr | Long palpebral fissure, eversion of third lateral, hypertelorism, epicanthus | Arched eyebrow, thinning of lateral third | Malformed and reared ear | Broad nasal root, bulbous nasal tip | Dysodontiasis, maxillary odontoma | High-arched palate |  | Yes | Thick hair | Clinodactyly (V) |
| P2 | M | 22 | *KMT2D* | Frameshift | *De novo* | 47 | c.14592dupG | p.(Pro4865Alafs*48) | Long palpebral fissure, palpebral eversion, epicanthus | Arched eyebrow, thinning of lateral third | Malformed and prominent ear | Broad nasal root, bulbous nasal tip, anteverse nostrils | Agenesias of right V inf |  | Thin upper lip, down lip corners | No |  | Generalized brachydactyly |
| P3 | F | 21 | *KDM6A* | Splicing | *De novo* | Intron 11-12 | c.975-1G>A (r.876_1320del ) | p.(Cys293Ilefs*26) | Long palpebral fissure, palpebral eversion, ptosis, strabismus (surgical correction) |  | Malformed ear | Broad nasal root, anteverse nostrils | Agenesias of upper lateral incisors |  | Thin upper lip |  | Low neck implant | Thumb hypoplasia, varus hallux, short metatarsus II-V |
| P4 | F | 23 | *KMT2D* | Nonsense | *De novo* | 31 | c.7903C>T | p.(Arg2635*) | Long palpebral fissure, hypertelorism, strabismus, ptosis | Arched eyebrow, thinning of lateral third | Malformed ear | Broad nasal root, short columella, depressed nasal tip | Agenesias of two upper lateral incisors | High-arched palate | Thin lips | Yes | Normal | Hands and feet brachydactyly |
| P5 | F | 23 | *KMT2D* | Frameshift | NA | 6 | c.705delA | p.(Glu237Serfs*24) | Long palpebral fissure, paplebral eversion hypertelorism, corneal leukoma | Arched eyebrow, thinning of lateral third | Malformed and prominent ear | Broad nasal root, short columella | Agenesias of two premolars, malocclusion | High-arched palate | Thin lips | Yes, microretrognathia |  | Hands brachy-clinodactyly (V) |
| P6 | F | 24 | *KMT2D* | Missense | *De novo* | 48 | c.15649T>C | p.(Trp5217Arg) | Long palpebral fissure, lower palpebral eversion | Thinning of lateral third | Malformed and prominent ear | Broad nasal root | Agenesias of upper lateral incisors, diastema | High-arched palate |  |  |  | Short metacarpus IV-V, sandal gap |
| P7 | M | 18 | *KMT2D* | Splicing | M | Intron 40-41 | r.13593_13671del | p.(Leu4532Serfs*7) | Long palpebral fissure, lower palpebral eversion, epicanthus, bulging optical disc in retinal nasal area |  | Prominent ear |  |  |  |  |  |  |  |
| P8 | M | 11 | *KMT2D* | Frameshift | *De novo* | 32 | c.8196delG | p.(Ser2733Valfs*24) | Long palpebral fissure, palpebral eversion hypertelorism, ptosis | Arched eyebrow, thinning of lateral third | Malformed and prominent ear | Short columella, depressed nasal root, anteverse nostrils |  | High-arched palate | Thin upper lip, down lip corners | Yes | High forehead | Brachy-clinodactyly (V) |
| P9 | M | 14 | *KMT2D* | Frameshift | *De novo* | 25 | c.5575delG | p.(Asp1859Thrfs*17) | Long palpebral fissure, palpebral eversion strabismus, ptosis, exophtalmos, epicanthus | Arched eyebrow, thinning of lateral third | Malformed and prominent ear | Short columella |  | High-arched palate | Thin lips | No | High forehead | Clinodactyly (V) |
| P10 | M | 10 | *KMT2D* | Nonsense | *De novo* | 39 | c.10750C>T | p.(Gln3584*) | Long palpebral fissure, lower palpebral eversion, myopia | Arched and thick eyebrow, thinning of lateral third | Malformed and prominent ear, low implant | Broad nasal root, short columella, depressed nasal tip, choanal stenosis | Eruptive cyst | High-arched palate, cleft | Thin upper lip | Yes |  | Hands campto-brachy-clinodactyly (IV-V) |
| P11 | F | 12 | *KMT2D* | Missense | *De novo* | 51 | c.16273G>A | p.(Glu5425Lys) | Long palpebral fissure, palpebral eversion, epicanthus | Thinning of lateral third | Malformed and prominent ear | Broad nasal root |  |  | Thin lips | Yes |  | Clinodactyly |
| P12 | F | 11 | *KMT2D* | Frameshift | *De novo* | 31 | c.6595delT | p.(Tyr2199Ilefs*65) | Long palpebral fissure, ptosis, exophtalmos | Thinning of lateral third | Malformed, prominent ear | Broad nasal root | Oligodontia | Palate cleft | Thin lips | No |  | V finger hypoplasia, feet middle and distal phalanx agenesis (II-IV-V) |
| P13 | M | 14 | *KMT2D* | Frameshift | NA | 48 | c.15031delG | p.(Glu5011Serfs*40) | Long palpebral fissure, palpebral eversion, strabismus, optical disc atrophy | Arched eyebrow | Malformed, prominent ear, low implant | Broad nasal root, short columella, depressed nasal tip, anteverse nostrils |  | Cleft surgical corrected | Thin lips |  | High forehead | Hands brachydactyly |
| P14 | M | 10 | *KMT2D* | Frameshift | NA | 11 | c.3161_3171delCGTTGAGTCCC | p.(Pro1054Hisfs*10) | Long palpebral fissure, palpebral eversion, ptosis | Arched eyebrow, thinning of lateral third | Malformed, prominent ear | Broad nasal root, short columella |  | High-arched palate | Thin lips with lip pit | Yes | High forehead |  |
| P15 | M | 15 | *KMT2D* | Nonsense | *De novo* | 31 | c.7891C>T | p.Gly2631* | Long palpebral fissure, eversion of third lateral | Arched and thick eyebrow, thinning of lateral third | Malformed and prominent ear | Broad nasal root, short columella, depressed nasal tip | Dysodontiasis | High-arched palate | Thin upper lip, down lip corners | Yes | High forehead | Campto-brachy-clinodactyly (V) |

**Table S2 Individual data of presently reported patients with Kabuki syndrome**

| **Patient** | **Fetal pads** | **Joint laxity/ hip/ knee/ foot** | **Vertebral** | **Pregnancy/ birth** | **Height** | **Weight** | **OFC** | **Endocrine** | **Intelligence/ behavior** | **EEG** | **MRI brain** | **Cardiac** | **Kidney/ urinary tract** | **Gastrointestinal** | **Hearing** | **Immunological/ Other** |
| --- | --- | --- | --- | --- | --- | --- | --- | --- | --- | --- | --- | --- | --- | --- | --- | --- |
| P1 | Yes |  | Scoliosis, C7 apophysis malformation | Full term, weight 3.700 kg | 3°-10° | 10° | 10°-25° | Autoimmune hypothyroidism, hypermenorrhea, micropolycystic ovary | Moderate disability | Sporadic pointed waves | Upper parietal gyrus reduction, enlarged ventricles |  | Mild pyelectasis |  |  | Angioma of fronto-nasal region, negative anti-tTG IgA and AGA IgA, decreased IgA, IgG, IgM |
| P2 | Yes | Joint laxity, valgus knee, flat foot | Lumbar scoliosis | 36 w, vaginal delivery, weight 2.850 kg | < 3° | 90°-97° | 10°-25° | Hypogonadotropic hypogonadism, hypogenitalism hyper-TSH, thyroid autoimmunity, GH deficiency, ginecomastia, cryptorchidism | Moderate disability (IQ . 49), hypotonia | Normal | Normal | Normal |  |  |  | Decreased IgG, normal IgA, IgM |
| P3 | Yes |  | Dorso-lumbar scoliosis | 36 w, IUGR, weight 2.500 kg, lenght 48 cm, OFC 32 cm | 25° | 90°-97° | 50° | Normal | Mild disability, aggressivity, soliloquy | Epilepsy, widespread paroxysm | Adenohypophysis hypoplasia, empty sella | Normal |  |  |  |  |
| P4 |  | Congenital hip dislocation, joint laxity, valgus knee, flat foot |  | Weight 3.230 kg, hypoglycemia on third day of life | < 5° | Obesity | 5° |  | Moderate disability (IQ . 47) |  |  | Bicuspid aortic valve, mild insufficiency | Pelvis ectasia, double kidney district, VUR |  | Bilateral conductive hearing loss | Decreased CD8, sacral angioma |
| P5 | Yes | Joint laxity | Dorso-lumbar scoliosis | Polidramnios, 37 w, weight 2.480 kg | 50°-75° | 25° | < 3° | Normal | Mild disability | Normal | Normal | VSD, aortic dilatation, abnormal retun into inferior vena cava |  |  | Chronic otitis (tympanoplasty) | Decreased IgA |
| P6 | Yes | Joint laxity |  | 39 w, weight 2.050 Kg | 10° | obesity | < 1 SD | GH deficiency | Intellectual disability | Epilepsy pharmacologically trated (lamotrigine), dizziness, hypotonia |  |  |  | Normal | Sensorineural and conductive hearing loss | Mottled and hypopigmented skin, hypoplastic nails |
| P7 | Yes |  |  | Weight 2.850 kg, lenght 50 cm, OFC 34 cm | < 3° | >90° | 58 cm | Orchidopexy at age 11, normal thyroid | Visual hallucinations, post-traumatic stress disorder, no intellectual disability (WPPSI-III IQ 80 age 2y+7 m) |  | Normal | Pericardial detachment | Renal cyst, enuresis |  |  | Polyserositis, decreased IgA, IgG, IgM, normal CD4/CD8, normal CD3 |
| P8 | Yes |  |  | Polidramnios, full term, weight 3.200 kg, | 3° | 25°-50° | 10° | Hyper-TSH, thyroid autoimmunity | Mild disability |  |  | VSD, ASD, AoCa, PDA |  |  |  | Cutaneus angiomas (neck and sacral area), decreased IgA, IgG, normal IgM |
| P9 | Yes | Valgus knee, flat foot |  | Polidramnios | < 5° |  | < 3° | Normal thyroid | Intellectual disability | Anormal EEG | Ischemia outcomes | VSD, AoCa |  |  |  | Negative anti-tTG IgA, decreased IgA, normal IgG, IgM, normal CD4/CD8, normal CD3 |
| P10 | Yes |  | L3 vertebral cleft | Polidramnios, weight 3.140, lenght 49 cm | 3°-10° | 50° | 10°-25° | Autoimmune hypothyroidism, cryptorchidism | Intellectual disability |  |  | VSD (surgical corrected), ASD, PDA | Double kidney district, renal ectopia |  |  | Left pulmonary artery hypoplasia, thymic ectopia, decreased IgA, IgG, IgM |
| P11 | Yes | Joint laxity, flat foot type III, mild hip dislocation | Scoliotic attitude | Weight 2.900 kg, lenght 48 cm | 25°-50° | 25°-50° | 10°-25° | Premature telarca, normal thyroid | Delayed speech, IQ 83 | Central pointed waves | Normal | Mitral insufficiency, mild apical hypertrophy |  |  | Conductive hearing loss, tympanic membrane perforation | Angioma of palpebral and frontal region, recurrent infections |
| P12 | Yes | Generalized joint laxity | L3 vertebral cleft | IUGR, polidramnios | < 5° | < 5° | < 3° | Premature telarca, hypoglycemia, hyperinsulinism | Intellectual disability |  | Enlarged lateral ventricles | VSD, PFO | Renal cyst, renal hypoplasia |  | Severe conductive hearing loss | Normal CD4/CD8, normal CD3 |
| P13 | Yes |  | Scoliosis | 37 w, weight 2.800 kg | < 3° | < 3° | < 3° | Delayed bone age, cryptorchidism | Moderate (IQ 43), then mild disability (IQ 61) |  |  | PFO |  |  | Chronic otitis |  |
| P14 | Yes |  | Butterfly vertebra | 37 w, 2.400 kg, microcephaly | 3°-10° | 10°-25° | < 3° | Hypoglycemia, cryptorchidism, no thyroid autoimmunity | Mild disability |  | Corpus callosum dysmorphism | VSD, AoCa (surgical corrected) |  |  |  | Inguinal hernia, recurrent infections, bronchial isomerism, bronchiectasis, decreased IgA, IgG, IgM, normal CD4/CD8, normal CD3 |
| P15 | Yes | Joint laxity | Dorso-lumbar scoliosis |  | <3° | <3° | 3°-10° | GH deficit | Mild disability |  | Pituitary microadenoma | Aortic valve displasy | Renal ectopia, fused kidney | Normal |  | Negative IgA TTG and AGA, normal IgG, IgA and IgM values |

OFC, occipitofrontal circumference; SD, standard deviation; IQ, intelligence quotient;VSD, ventricular septal defect; ASD, atrial septal defect; AoCa, aortic coarctation; PDA, persistent ductus arteriosus; PFO, patent foramen ovale; VUR, vescicoureteric reflux;  anti-tTG, transglutaminase antibodies; AGA, antigliadin antibodies
